# Identification of gene pathways implicated in Alzheimer's disease using longitudinal imaging phenotypes with sparse regression^☆^

**DOI:** 10.1016/j.neuroimage.2012.08.002

**Published:** 2012-11-15

**Authors:** Matt Silver, Eva Janousova, Xue Hua, Paul M. Thompson, Giovanni Montana, The Alzheimer's Disease Neuroimaging Initiative

**Affiliations:** a Statistics Section, Department of Mathematics, Imperial College London, UK; b Institute of Biostatistics and Analyses, Masaryk University, Brno, Czech Republic; c Laboratory of Neuro Imaging, Department of Neurology, UCLA School of Medicine, Los Angeles, CA, USA

**Keywords:** Alzheimer's disease, Imaging genetics, Atrophy, Gene pathways, Sparse regression

## Abstract

We present a new method for the detection of gene pathways associated with a multivariate quantitative trait, and use it to identify causal pathways associated with an imaging endophenotype characteristic of longitudinal structural change in the brains of patients with Alzheimer's disease (AD). Our method, known as pathways sparse reduced-rank regression (PsRRR), uses group lasso penalised regression to jointly model the effects of genome-wide single nucleotide polymorphisms (SNPs), grouped into functional pathways using prior knowledge of gene–gene interactions. Pathways are ranked in order of importance using a resampling strategy that exploits finite sample variability. Our application study uses whole genome scans and MR images from 99 probable AD patients and 164 healthy elderly controls in the Alzheimer's Disease Neuroimaging Initiative (ADNI) database. 66,182 SNPs are mapped to 185 gene pathways from the KEGG pathway database. Voxel-wise imaging signatures characteristic of AD are obtained by analysing 3D patterns of structural change at 6, 12 and 24 months relative to baseline. High-ranking, AD endophenotype-associated pathways in our study include those describing insulin signalling, vascular smooth muscle contraction and focal adhesion. All of these have been previously implicated in AD biology. In a secondary analysis, we investigate SNPs and genes that may be driving pathway selection. High ranking genes include a number previously linked in gene expression studies to *β*-amyloid plaque formation in the AD brain (*PIK*3*R*3, *PIK*3*CG*, *PRKCA* and *PRKCB*), and to AD related changes in hippocampal gene expression (*ADCY2*, *ACTN1*, *ACACA*, and *GNAI1*). Other high ranking previously validated AD endophenotype-related genes include *CR1*, *TOMM40* and *APOE*.

## Highlights

► New sparse regression method to find pathways associated with a multivariate trait ► Imaging phenotypes describe longitudinal structural change in Alzheimer's disease. ► Insulin signalling and vascular smooth muscle contraction pathways highly ranked ► Top genes include PIK3R3 PIK3CG PRKCA/B ADCY2 ACTN1 ACACA GNAI1 CR1 TOMM40 and APOE. ► All these genes previously implicated in different aspects of AD biology.

## Introduction

A growing list of genetic variants has now been associated with greater susceptibility to develop early and late-onset Alzheimer's disease (AD), with the *APOEϵ*4 allele consistently identified as having the greatest effect (for an up to date list see www.alzgene.org). Recently, case–control susceptibility studies have been augmented by studies using neuroimaging phenotypes. The rationale here is that the use of heritable imaging signatures (endophenotypes) of disease may increase the power to detect causal variants, since gene effects are expected to be more penetrant at this level (Meyer-Lindenberg and Weinberger, 2006). This ‘imaging-genetic’ approach has been used to identify genes associated with a range of AD-associated imaging phenotypes including measures of hippocampal volume (Stein et al., 2012), cortical thickness (Burggren et al., 2008) and longitudinal, structural change (Vounou et al., 2011).

AD is a moderate to highly heritable condition, yet as with many common heritable diseases, association studies have to date identified gene variants explaining only a relatively modest amount of known AD heritability (Braskie et al., 2011). One approach to uncovering this ‘missing heritability’ is motivated by the observation that in many cases disease states are likely to be driven by multiple genetic variants of small to moderate effect, mediated through their interaction in molecular networks or pathways, rather than by the effects of a few, highly penetrant mutations (Schadt, 2009). Where this assumption holds, the hope is that by considering the joint effects of multiple variants acting in concert, pathways genome-wide association studies (PGWAS) will reveal aspects of a disease's genetic architecture that would otherwise be missed when considering variants individually (Fridley and Biernacka, 2011; Wang et al., 2010). Another potential benefit of the PGWAS approach is that it can help to elucidate the mechanisms of disease by providing a biological interpretation of association results (Cantor et al., 2010). In the case of AD for example, an understanding of the underlying mechanisms by which gene mutations impact disease aetiology may play an important role in the translation of basic AD biology into therapy and patient care (Sleegers et al., 2010).

In this paper, we present the first PGWAS method that is able to accommodate a multivariate quantitative phenotype, and apply our method to a pathway analysis of the ADNI cohort, comparing genome-wide single nucleotide polymorphism (SNP) data with voxel-wise tensor-based morphometry (TBM) maps describing longitudinal structural changes that are characteristic of AD. In this study we map SNPs to pathways from the KEGG pathway database, a curated collection of functional gene pathways representing current knowledge of molecular interaction and reaction networks (http://www.genome.jp/kegg/pathway.html). Our method is however able to accommodate alternative sources of information for the grouping of SNPs and genes, for example using gene ontology (GO) terms, or information from protein interaction networks (Jensen and Bork, 2010; Wu et al., 2010).

The use of high-dimensional endophenotypes in imaging genetic studies has become increasingly commonplace, since it enables the voxel-wise mapping of genetic effects across the brain (Thompson et al., 2010). Previous work has demonstrated that a sparse reduced-rank regression (sRRR) approach that exploits the multivariate nature of the phenotype can be more powerful than a mass-univariate linear modelling approach in which each phenotype is regressed against each SNP (Vounou et al., 2010). Furthermore, multivariate, high-dimensional phenotypes have also been shown to offer an increased signal to noise ratio over low dimensional or univariate phenotypes, provided that uninformative voxels that are not characteristic of the disease under study are removed (Vounou et al., 2011). In this study we use a high-dimensional phenotype describing structural change relative to baseline over three time points in subjects with AD, and in healthy controls. From this we extract an imaging endophenotype that is highly characteristic of AD in our sample by using a stringent statistical threshold to exclude voxels that do not discriminate between AD and CN. Our main objective here is not to build a robust statistical classifier for AD, but instead to produce a quantitative phenotype having maximal sample variability between AD and CN for the subsequent gene mapping stage of our analysis.

Many existing PGWAS methods, such as GenGen (Wang et al., 2009) and ALLIGATOR (Holmans et al., 2009) rely on univariate statistics of association, whereby each SNP in the study is first independently tested for association with a univariate quantitative or dichotomous (case–control) phenotype. SNPs are assigned to pathways by mapping them to adjacent genes within a specified distance, and individual SNP or gene statistics are then combined across each pathway to give a measure of pathway significance, corrected for multiple testing. Methods must also account for the potentially biasing effects of gene and pathway size and linkage disequilibrium (LD), and this is generally done through permutation. A potential disadvantage of these methods is that each SNP is considered separately at the first step, with no account taken of SNP–SNP dependencies. In contrast, a multilocus or multivariate model that considers all SNPs simultaneously may characterise SNP effects more accurately by aiding the identification of weak signals while diminishing the importance of false ones (Hoggart et al., 2008).

In earlier work we developed a multivariate PGWAS method for identifying pathways associated with a single quantitative trait (Silver and Montana, 2012). We used a sparse regression model – the group lasso – with SNPs grouped into pathways. We demonstrated in simulation studies using real SNP and pathway data, that our method showed high sensitivity and specificity for the detection of important pathways, when compared with an alternative pathway method based on univariate SNP statistics. Our method showed the greatest relative gains in performance where marginal SNP effect sizes are small. Here we extend our previous model to accommodate the case of a multivariate neuroimaging phenotype. We do this by incorporating a group sparsity constraint on genotype coefficients in a multivariate sparse reduced-rank regression model, previously developed for the identification of single causal variants (Vounou et al., 2010). Our proposed ‘pathways sparse reduced-rank regression’ (PsRRR) algorithm incorporates phenotypes and genotypes in a single model, and accounts for potential biasing factors such as dependencies between voxels and SNPs using an adaptive, weight-tuning procedure.

To the best of our knowledge, few other multilocus methods for the identification of biological pathways currently exist. The GRASS method (Chen et al., 2010) and the method proposed by Zhao et al. (2011) use sparse regression techniques to measure pathway significance. These methods are currently implemented for case–control data only, and are unable to accommodate a multivariate phenotype. Each method makes different assumptions about the distribution of important SNPs and genes affecting the phenotype. GRASS assumes sparsity at the SNP level within each pathway gene, while Zhao's method assumes sparsity at the gene level. In contrast, our PsRRR method assumes sparsity only at the pathway level (although we subsequently perform SNP and gene selection as a second step in selected pathways). As such, each method is expected to perform differently, depending on the ‘true’ distribution of causal SNPs and genes. GRASS and Zhao's methods also use a pre-processing dimensionality reduction step on SNPs within each gene using PCA. While this has been shown to be advantageous in certain circumstances (Wang and Abbott, 2008), we elect to retain original SNP genotypes in our model, since this facilitates sparse SNP selection. A further distinguishing feature of our method is that we include all pathways together in a single regression model. By doing this we hope to gain a better measure of the relative importance of different pathways, by ensuring that they compete against each other.

The article is presented as follows. We begin in the Imaging data section with a description of the voxel-wise TBM maps used in the study, and in the Phenotype extraction section we outline how we use these maps to generate an imaging signature characteristic of structural change in AD, that is able to discriminate between AD patients and controls. In the Genotype data section we describe the genotype data used in the study, together with quality control procedures, and in the SNP to pathway mapping section we explain how this genotype data is mapped to gene pathways. The theoretical underpinnings of the PsRRR method are described in the Pathways sparse reduced-rank regression section. We explain our method for ranking AD-associated pathways, SNPs and genes using a resampling procedure in the Pathway, gene and SNP ranking section, and discuss our strategies for addressing the significant computational challenge of fitting a regression-based model with such high dimensional datasets in the Computational issues section. Pathway, SNP and gene ranking results are presented in the Results section, and we conclude with a Discussion.

## Materials and methods

Imaging and genotype data used in this study were obtained from the Alzheimer's Disease Neuroimaging Initiative (ADNI) database (adni.loni.ucla.edu). The ADNI was launched in 2003 by the National Institute on Aging (NIA), the National Institute of Biomedical Imaging and Bioengineering (NIBIB), the Food and Drug Administration (FDA), private pharmaceutical companies and non-profit organisations, as a 5-year public–private partnership. The primary goal of ADNI has been to test whether serial magnetic resonance imaging (MRI), positron emission tomography (PET), other biological markers, and clinical and neuropsychological assessment can be combined to measure the progression of mild cognitive impairment (MCI) and early AD. Determination of sensitive and specific markers of very early AD progression is intended to aid researchers and clinicians to develop new treatments and monitor their effectiveness, as well as lessen the time and cost of clinical trials.

### Imaging data

Longitudinal brain MRI scans (1.5 T) were downloaded from the ADNI public database (http://www.loni.ucla.edu/ADNI/Data/). Serial brain MRI scans (N = 3512; see Table 1) were analysed from 200 probable AD patients and 232 healthy elderly controls (CN). AD subjects were scanned at screening and followed up at 6, 12, and 24 months, CN subjects at 6, 12, 24 36 and 48 months. All subjects were scanned with a standardised 1.5 T MP-RAGE protocol developed for ADNI (Jack et al., 2008). The typical acquisition parameters were repetition time (TR) of 2400 ms, minimum full echo time (TE), inversion time (TI) of 1000 ms, flip angle of 8, 24 cm field of view, 192 × 192 × 166 acquisition matrix in the *x* -, *y* -, and *z‐*dimensions, yielding a voxel size of 1.25 × 1.25 × 1.2 mm^3^, and later reconstructed to 1 mm isotropic voxels. Image correction steps included gradwarp (Jovicich et al., 2006), B1-correction (Jack et al., 2008), N3 bias field correction (Sled et al., 1998), and phantom-based geometrical scaling (Gunter et al., 2006).

Linear registration (9-parameter) was used to align the longitudinal scan series of each subject and then the mutually aligned time-series was registered to the International Consortium for Brain Mapping template (ICBM-53) (Mazziotta et al., 2001). Brainmasks that excluded the skull, other non-brain tissues, and the image background were generated automatically using a parameter-less robust brain extraction tool (ROBEX) (Iglesias et al., 2011).

Individual Jacobian maps were created to estimate 3D patterns of structural brain change over time by warping the skull-stripped, globally registered and scaled follow-up scan to match the corresponding screening scan. We used a non-linear, inverse consistent, elastic intensity-based registration algorithm (Leow et al., 2005), which optimises a joint cost function based on mutual information (MI) and the elastic energy of the deformation. Colour-coded maps of the Jacobian determinants were created to illustrate regions of ventricular/CSF expansion (i.e., with det *J*(*r*) > 1), or brain tissue loss (i.e., with det *J*(*r*) < 1) (Ashburner and Friston, 2003; Chung et al., 2001; Freeborough and Fox, 1998; Riddle et al., 2004; Thompson et al., 2000; Toga, 1999) over time. These longitudinal maps of tissue change were also spatially normalised across subjects by nonlinearly aligning all individual Jacobian maps to an average group template known as the minimal deformation target (MDT), for regional comparisons and group statistical analyses.

The study was conducted according to the Good Clinical Practice guidelines, the Declaration of Helsinki and U.S. 21 CFR Part 50-Protection of Human Subjects, and Part 56-Institutional Review Boards. Written informed consent was obtained from all participants before experimental procedures, including cognitive tests, were performed.

### Phenotype extraction

We include 253 individuals (99 AD, 154 CN) with longitudinal maps at all three time points (6, 12 and 24 months), who have also been genotyped by ADNI. Other time points are excluded because of missing observations.

To maximise the power to detect causal pathways, we seek a phenotype which is highly representative of those structural changes in the brain that are characteristic of AD. One way to do this is to use prior knowledge on regions of interest (ROI) to extract a univariate quantitative measure as a disease signature (Potkin et al., 2009). We instead use a voxel-wise, data-driven approach to produce a multivariate disease signature that may present a stronger signal for the detection of genetic effects (Vounou et al., 2011).

A previous imaging genetic study on the same ADNI cohort measured structural change relative to baseline at a single time point only. In that study an AD-specific phenotype was produced using a sparse linear classifier to select a subset of voxels that minimised the CN/AD classification error (Vounou et al., 2011). In the present study where we incorporate two additional timepoints, we instead begin by fitting a linear regression with an intercept term, where the dependent variable is the voxel value (change relative to baseline at screening), and the independent variable is time. The regression coefficient for the slope thus gives a summary measure of tissue change over time at each voxel. To obtain a phenotype that is maximally discriminative between CN and AD in our sample, we remove all voxels where the difference in the slopes is not significantly different from zero, by performing an analysis of variance (ANOVA), with sex and age as covariates. Finally we select the most discriminative voxels whose ANOVA p-values exceed a level of 0.05, with a Bonferroni correction for multiple testing. Once again, the use of an ultra-conservative significance threshold ensures that our phenotypic disease signature is maximally discriminative between CN and AD in our sample. The final set of phenotypes used in the study then corresponds to the voxel-wise slope coefficients for all 253 subjects at the selected voxels, corrected for sex and age.

### Genotype data

Genotypes for the 464 subjects in the study were obtained from the ADNI database. ADNI genotyping is performed using the Human610-Quad Bead-Chip, which includes 620,901 SNPs and copy number variations (see Saykin et al., 2010 for details). SNPs defining the *APOE*ϵ4 variant are not included in the original genotyping chip, but have been genotyped separately by ADNI. These were added to the final genotype dataset. Subjects were unrelated, and all of European ancestry, and passed screening for evidence of population stratification using the procedure described in Stein et al. (2010). We included only autosomal SNPs in the study (78,874 markers excluded), and additionally excluded SNPs with a genotyping rate < 95 % (42,680 SNPs), a Hardy–Weinberg equilibrium p-value < 5 × 10^− 7^ (873 SNPs), and a minor allele frequency < 0.1 (64,204 SNPs). Finally, since our method does not allow for missing SNP minor allele counts, missing genotypes were imputed (see Vounou et al., 2011 for details). 434,271 SNPs remained after all SNP filtering steps described above.

### SNP to pathway mapping

Our SNP mapping procedure rests on the extraction of prior information from a pathway database that provides curated lists of genes, mapped to functional networks or pathways. Pathway databases such as those provided by KEGG (http://www.genome.jp/kegg/pathway.html), Reactome (http://www.reactome.org/) and Biocarta (http://www.biocarta.com/) typically classify pathways across a number of functional domains, for example apoptosis, cell adhesion or lipid metabolism; or crystallise current knowledge on specific disease-related molecular reaction networks.

**Fig. 1:**
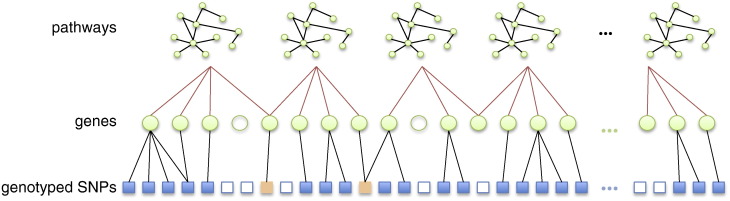
Schematic illustration of the SNP to pathway mapping process. (i) Known genes (green circles) are mapped to pathways using information on gene–gene interactions (top row), obtained from a gene pathway database. Many genes do not map to any known pathway (unfilled circles). Also, some genes may map to more than one pathway. (ii) Genes that map to a pathway are in turn mapped to genotyped SNPs within a specified distance. Many SNPs cannot be mapped to a pathway since they do not map to a mapped gene (unfilled squares). Note SNPs may map to more than one gene. Some SNPs (orange squares) may map to more than one pathway, either because they map to multiple genes belonging to different pathways, or because they map to a single gene that belongs to multiple pathways.

**Fig. 2:**
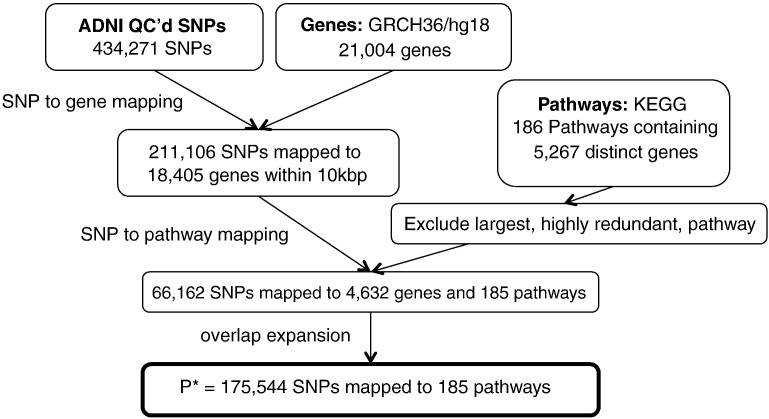
Mapping SNPs to pathways.

Starting with a list of all genes that map to at least one pathway in the database, we assign SNPs to genes within a specified distance, upstream or downstream of the gene in question, and thence to pathways. This process is illustrated schematically in Fig. 1. For our AD pathway study, we proceed as follows. A list of 21,004 human gene chromosomal locations, corresponding to human genome assembly GRCH36 was obtained using Ensembl's BIOMART API (www.biomart.org). SNPs were then mapped to any gene within 10 kilo base pairs, upstream or downstream of the gene in question. This resulted in 211,106 SNPs being mapped to 18,405 genes. While the majority of known genes did map to at least one SNP in our study, approximately half of the SNPs passing QC were not located within 10 kbp of a known gene. For pathway mapping, we used the KEGG canonical pathway gene sets obtained from the Molecular Signatures Database v3.0 (http://www.broadinstitute.org/gsea/msigdb/index.jsp), which contains 186 gene sets, which map to a total of 5267 distinct genes, with many genes mapping to more than one pathway. Note that only around 25 % of all known genes map to a pathway in this dataset. We map all SNPs within 10 kilo base pairs of one or more of the 5267 pathway-mapped genes to the pathway(s) concerned. Finally, we exclude the largest pathway, by number of mapped SNPs, (‘Pathways in Cancer’) that is highly redundant, in that it contains multiple other pathways as subsets. This results in 66,162 SNPs mapped to 4425 genes and 185 pathways (see Fig. 2).

**Fig. 3:**
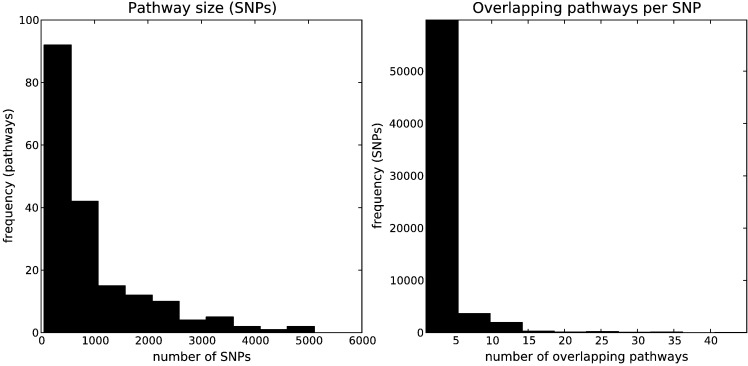
*Left*: Pathway sizes. Distribution of KEGG pathways, by the number of ADNI SNPs that they map to. *Right*: SNP overlaps. Distribution of ADNI SNPs, by the number of pathways that they map to. SNPs map to multiple pathways either because they map to a gene that belongs to more than one pathway, or because they map to more than one gene belonging to more than one pathway.

The distribution of pathway sizes in terms of the number of SNPs that they map to is illustrated in Fig. 3 (left). Pathway sizes range from 57 to 5111 SNPs (mean 949). The distribution of overlapping SNPs, that is the number of pathways to which each SNP is mapped, is illustrated in Fig. 3 (right). This ranges from 1 to 45 pathways (mean 2.65).

Note that following the above procedure, some genes previously implicated in AD studies do not map to any pathways, and thus are not included in the analysis. For example, in this study, 12 out of 30 genes highlighted in the review by Braskie et al. (2011) are mapped to pathways. The remaining 18 genes are excluded because they do not feature in any KEGG pathway. Also note that since SNPs are mapped to all genes within a range of 10 kbp, AD implicated SNPs may map to more than one gene, and its corresponding pathway(s). This is the case for example with a number of SNPs mapping to the APOE and TOMM40 genes. This information is summarised in Table 2.

### Pathways sparse reduced-rank regression

We consider the problem of identifying gene pathways associated with a multivariate quantitative trait (MQT) or phenotype, $Y\in R^{Q}$. The observed values for phenotype *q*, measured for *N* unrelated individuals, are arranged in an (*N* × 1) response vector **y**_*q*_, and the *Q* phenotypes are arranged in an (*N* × *Q*) response matrix **Y** = (**y**_1_,…,**y**_*Q*_). We assume that minor allele counts for *P* SNPs are recorded for all individuals, and denote by *x*_*ij*_ the minor allele count for SNP *j* on individual *i*. These are arranged in an (*N* × *P*) genotype design matrix **X**. We additionally assume all phenotypes and genotypes are mean centred, and that SNP genotypes are standardised to unit variance, so that $\sum_{i}x_{\mathrm{ij}}^{2}=1$, for *j* = 1,…, *P*.

If we denote by **C** = (**C**_1_,…, **C**_*Q*_), a (*P* × *Q*) matrix of regression coefficients, then we can model the multivariate response as

(1) Y=XC+E

where **E** is an (*N* × *Q*) matrix of error terms. A least squares estimate for **C** may be obtained by generalising the multiple least squares optimisation to include a multivariate response, that is by minimising the residual sum of squares

(2) $${\text{M}}^{\mathrm{MMLR}}=\text{Tr}\left\{\left(Y-XC\right){\left(Y-XC\right)}^{\prime }\right\}\text{.}$$

Where *N* > *P*, and the design matrix **X** is of full rank, the least squares estimates are given by $\hat{C}={\left(X^{\prime }X\right)}^{-1}X^{\prime }Y$. Note that the (*P* × 1) column vectors ${\hat{C}}_{1},\ldots ,{\hat{C}}_{Q}$ of $\hat{C}$ are just the least squares estimates of the regression of each **y**_*q*_ on **X**, that is

(3) $${\hat{C}}_{q}=\arg\min_{C_{q}}{\left\|y_{q}-{XC}_{q}\right\|}_{2}^{2}\;q=1\text{,…, }Q$$

where ‖ ⋅ ‖_2_ denotes the $\ell_{2}$ (Euclidean) norm.

For high-dimensional datasets, such as those typically found in genomics, this model is unsuitable for a number of reasons. Firstly, *P* ≫ *N*, so that $X^{\prime }X$ is singular and thus not invertible and the estimates ${\hat{C}}_{q}$ are not uniquely defined. Even where *P* < *N*, for example in a candidate gene study, LD or equivalently near multi-collinearity between predictors means that $X^{\prime }X$ is nearly singular, resulting in inflated variance in SNP coefficient estimates. Furthermore, the estimation (3) is equivalent to performing *Q* independent regressions, and takes no account of the multivariate nature of **Y**. Ideally, we would like to exploit this in our estimation procedure to boost power (Breiman and Friedman, 1997; Vounou et al., 2010).

These limitations are addressed in *reduced-rank regression* (RRR), (Izenman, 2008), by restricting the rank of the coefficient matrix **C**. Specifically we impose the constraint that **C** has rank *r* < min(*P*, *Q*), and rewrite **C** as **C** = **BA**, where **A** and **B** both have (full) rank *r*. The reduced rank form of Eq. (1) is then given by

(4) Y=XBA+E

where **B** and **A** are (*P* × *r*) and (*r* × *Q*) matrices of regression coefficients respectively relating to genotypes and phenotypes. This model has the interesting interpretation of exposing *r* hidden or *latent factors*, which capture the major part of the relationship between **Y** and **X**. If we denote by **B**_(*k*)_, the *k*th column of **B**, then we see that the products ${XB}_{\left(k\right)},k=1\text{,…, }r$, represent *r* linear combinations of the *P* predictor variables. Similarly, the *r* row vectors, **A**_(*k*)_, *k* = 1,…, *r*, represent the transformation of each of these back to the dimensions of **Y**, so that they can predict the response. The linear combinations ${XB}_{\left(k\right)}$ and ${YA^{\prime }}_{\left(k\right)}$ thus represent a reduced set of *r* (latent) factors that capture the relationship between response and predictors, reduced in the sense that this set has dimensionality *r* < min(*P*, *Q*).

We consider the rank-1 RRR model which captures the first, main set of genotype and phenotype latent factors describing the association between **X** and **Y**. With *r* = 1, we rewrite Eq. (4) as

(5) Y=Xba+E

where **b** and **a** are (*P* × 1) and (1 × *Q*) coefficient vectors respectively relating to genotypes and phenotypes. Least squares estimates for $\hat{b}$ and $\hat{a}$ are then obtained by minimising the rank-1 equivalent of Eq. (2),

(6) $${\text{M}}^{RR_{1}R}=\text{Tr}\left\{\left(Y-Xba\right)\Gamma {\left(Y-Xba\right)}^{\prime }\right\}$$

where *Γ* is a given (*q* × *q*) positive definite matrix of weights. The choice of *Γ* reflects how we deal with correlation between the responses **y**_1_,…, **y**_*q*_ in the least squares optimisation. Such correlations can be exploited by setting *Γ* to be the inverse of the estimated covariance of the responses. In the context of imaging genetics for example, where a voxel-wise multivariate response may be derived from structural MRI, spatial correlations between phenotypes are expected in part to reflect common genetic variation. However, the calculation of the inverse ${\left(Y^{\prime }Y\right)}^{-1}$ is computationally very intensive, and is in any case likely to be inaccurate for small sample sizes, so we instead use the simplifying approximation *Γ* = **I**_*q*_, effectively assuming the responses to be uncorrelated (Vounou et al., 2010, 2011).

We now turn to the case where all *P* SNPs may be mapped to *L* groups, $G_{l}\subset \left\{1\text{,…,}\;P\right\}$, *l* = 1, …, *L*, for example by mapping SNPs to gene pathways (see the SNP to pathway mapping section). We begin by assuming that pathways are disjoint or non-overlapping, that is $G_{l}\cap G_{l^{\prime }}\neq \emptyset$ for any *l* ≠ *l*′. We denote the rank-1 vector of SNP regression coefficients by **b** = (*b*_1_,…, *b*_*P*_). We additionally denote the matrix containing all SNPs mapped to pathway $G_{l}$ by **X**_*l*_ = (*X*_*l*_1__, *X*_*l*_2__,…, *X*_*S*_*l*__), where *X*_*j*_ = (*x*_1*j*_, *x*_2*j*_,…, *x*_*Nj*_)′, is the column vector of observed SNP minor allele counts for SNP *j*, and *S*_*l*_ is the number of SNPs in $G_{l}$. Finally, we denote the corresponding vector of SNP coefficients by **b**_*l*_ = (*b*_*l*_1__, *b*_*l*_2__,…, *b*_*S*_*l*__).

**Fig. 4:**
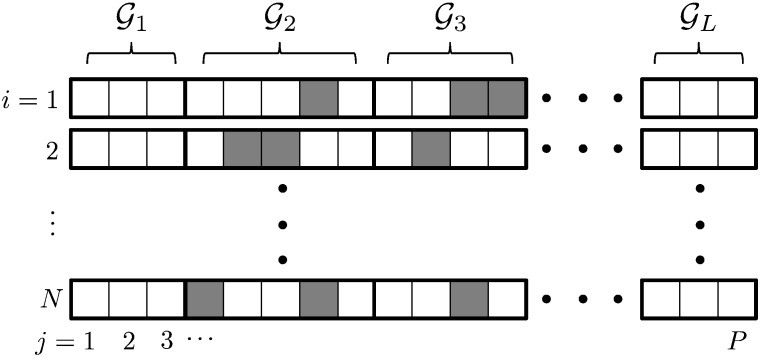
Group-sparse distribution of causal SNPs. The set $S\subset \left\{1\text{,…, }P\right\}$ of causal SNPs influencing the phenotype are represented by boxes that are shaded grey. Causal SNPs are assumed to occur within a set C of causal pathways. Here $C=\left\{2,3\right\}$. Note that the particular distribution of causal SNPs may vary for each individual, *i* = 1,…, *N*. The group sparsity assumption is that $\left|C\right|\ll L$.

In general, where *P* is large, we expect only a small proportion of SNPs to be ‘causal’, in the sense that they exhibit phenotypic effects. We further assume that causal SNPs will tend to be enriched within functional groups, or gene pathways. This latter assumption is illustrated schematically in Fig. 4, where causal SNPs (marked in grey) tend to accumulate within a small number of causal pathways, while the majority of pathways contain no causal SNPs. A model that generates such a sparsity pattern is said to be *group-sparse*, in that SNPs affecting **Y** are to be found in a set $C\subset \left\{1\text{,…, }L\right\}$ of causal gene pathways (groups), with |C|≪L, where $\left|C\right|$ denotes the cardinality of C. We seek a parsimonious model that is able to identify this set, C, of causal pathways, by imposing a group-sparsity constraint on the estimated SNP coefficient vector, **b**.

In *sparse reduced-rank regression* (sRRR) (Vounou et al., 2010, 2011), sparse estimates for genotype and/or phenotype coefficient vectors are obtained by imposing a regularisation penalty on **b** and/or **a** respectively. Apart from the benefits of model parsimony, enforcing a sparsity constraint on **b** also allows us to deal with the *P* ≫ *N* case, and with multicollinearity between predictors. In our proposed ‘pathways sparse reduced-rank regression’ (PsRRR) model, the required group sparsity pattern is obtained by imposing an additional group lasso penalty (Yuan and Lin, 2006) on Eq. (6). Group-sparse solutions to the rank-1 RRR model (5) are then obtained by minimising the following penalised least squares problem

(7) $${\text{M}}^{PsRR_{1}R}=\frac{1}{2}\text{Tr}\left\{\left(Y-Xba\right){\left(Y-Xba\right)}^{\prime }\right\}+\text{λ}\sum_{l=1}^{L}w_{l}{\left\|b_{l}\right\|}_{2}$$

with respect to **b** and **a**. Eq. (7) corresponds to an ordinary least squares (OLS) optimisation, but with an additional group-wise penalty whose size depends on ||**b**_*l*_||_2_, *l* = 1,…, *L*, a regularisation parameter λ, and an additional group weighting parameter *w*_*l*_ that can vary from group to group. Depending on the value of λ, this penalty has the effect of setting multiple pathway SNP coefficient vectors, **b**_*l*_ = **0**, *l* ⊂ {1,…, *L*}, thereby enforcing group sparsity. Pathways with non-zero coefficient vectors form the set $\hat{C}$ of *selected* pathways, so that

$$\hat{C}\left(\text{λ}\right)=\left\{l:b_{l}\neq 0\right\}\text{.}$$

Expanding Eq. (7), and noting that the first term $YY^{\prime }$ does not depend on **b** or **a**, solutions satisfy

(8) $$\hat{b},\hat{a}=\underset{b,a}{\arg\min}\;\left\{\frac{1}{2}\left(-2aY^{\prime }Xb+aa^{\prime }b^{\prime }X^{\prime }Xb\right)+\text{λ}\sum_{l=1}^{L}w_{l}{\left\|b_{l}\right\|}_{2}\right\}\text{.}$$

For fixed **a**, this penalised least squares problem equates to a convex optimisation in **b**, and is thus amenable to solution using coordinate descent (Friedman et al., 2007). A global solution can then be obtained by iteratively estimating one coefficient vector (**b** or **a**), while holding the other fixed at its current value, until convergence (Chen and Chan, 2012).

Thus, for fixed **b** and λ, and with the additional constraint that $bb^{\prime }=1$, we estimate $\hat{a}$ as

$$\hat{a}=\underset{a}{\arg\min}\;\left\{\frac{1}{2}\left(-2aY^{\prime }Xb+aa^{\prime }b\prime X^{\prime }Xb\right)+\text{λ}\sum_{l=1}^{L}w_{l}{\left\|b_{l}\right\|}_{2}\right\}\text{.}$$

Differentiating and setting to zero gives

$$\hat{a}=\frac{{\hat{b}}^{\prime }X^{\prime }Y}{{\hat{b}}^{\prime }X^{\prime }X\hat{b}}\text{.}$$

Similarly, for fixed **a**, and with the additional constraint that $aa^{\prime }=1$, we have

(9) $$\hat{b}=\underset{b}{\arg\min}\;\left\{\frac{1}{2}\left(-2aY^{\prime }Xb+b\prime X^{\prime }Xb\right)+\text{λ}\sum_{l=1}^{L}w_{l}{\left\|b_{l}\right\|}_{2}\right\}\text{.}$$

This is equivalent to a standard group lasso estimation problem with univariate response vector $Ya^{\prime }$. In an earlier work we describe a method, ‘Pathways Group Lasso with Adaptive Weights’ (P-GLAW), for solving this problem, specifically tailored to the situation where predictor variables are SNPs grouped into pathways (Silver and Montana, 2012). Here, we briefly recap key points of this method, and incorporate a number of extensions designed to accommodate a MQT in the context of PsRRR with coordinate descent.

The minimising function (9) is convex, and can be solved using block coordinate descent (BCD) (Friedman et al., 2010), an extension of coordinate descent to convex estimation with grouped variables. BCD rests on obtaining successive estimates, **b**_*l*_, for each pathway in turn, while keeping current estimates for all other pathways, **b**_*k*_, *k* ≠ *l*, constant, until a global minimum is obtained. For pathway $G_{l}\text{, }l=1\text{,…, }L$, estimates for each SNP coefficient, *b*_*j*_, *j* = *l*_1_,…, *l*_*S*_*l*__ are obtained through coordinate descent within the group. The group lasso estimation algorithm using BCD is presented in Box 1.

As λ increases, fewer groups (or pathways) are selected by the model (Box 1, step 5), while for selected pathways with **b**_*l*_ ≠ 0, estimated SNP coefficients, *b*_*j*_, *j* = *l*_1_,…, *S*_*l*_, tend to shrink towards zero (Box 1, step 11).

The full PsRRR estimation algorithm is presented in Box 2.

Note that we set the regularisation parameter, λ, to be a constant fraction (*γ*) of the maximal value, λ_*max*_, where no groups are selected by the model.

A key feature of our P-GLAW method is the need to accommodate the fact that pathways overlap, that is $G_{l}\cap G_{l^{\prime }}\neq \emptyset$ for some *l* ≠ *l*′, since SNPs may map to multiple pathways. To enable the independent selection of pathways, we instead require that groups are disjoint (Jacob et al., 2009). This is achieved through an expansion of the design matrix, **X**, formed from the column-wise concatenation of the *L* sub-matrices of size (*N* × *S*_*l*_), so that **X** = [**X**_1_, **X**_2_,…, **X**_*L*_]. This expanded **X** has dimensions (*N* × *P*^∗^), with $P^{*}=\sum_{l}\;S_{l}$. A corresponding expansion of the parameter vector, **b** = [**b**_1_′, **b**′_2_,…, **b**_*L*_′]′ is also required. The expansion of the design matrix enables the same SNP to be selected (or not selected) in one pathway, while remaining unselected (or selected) in another pathway to which it is mapped. Interaction effects between pathways arising from replicated SNPs will occur, but in simulation studies we have found that multiple interacting causal pathways may be selected by the model (Silver and Montana, 2012).

Another issue that we address is the problem of pathway selection bias, by which we mean the tendency of the group lasso to favour the selection of specific pathways, under the null, where no SNPs influence the phenotype. Such biases can arise for example from variations in the number of SNPs or genes in pathways, and varying patterns of dependence (LD) between SNPs within pathways. Under the null, with the regularisation parameter λ tuned so that a single pathway is selected, pathway selection probabilities should follow a uniform distribution, namely with probability *Π*_*l*_ = 1/*L*, for *l* = 1,…, *L*. However, where biasing factors are present, the empirical probability distribution, *Π*^∗^ will not be uniform. Our iterative weight tuning procedure works by applying successive adjustments to the pathway weight vector, **w** = (*w*_1_,…, *w*_*L*_), so as to reduce the difference, *d*_*l*_ = Π_*l*_^∗^(w) − Π_*l*_, between the unbiased and empirical (biased) distributions for each pathway. We begin with an initial weight vector, $w^{\left(0\right)}=\sqrt{S_{l}}$, which corrects for the biasing effect of group size in the group lasso model (Silver and Montana, 2012). At iteration *τ*, we compute the empirical pathway selection probability distribution Π_*l*_^∗^(w^(*τ*)^) over multiple model fits with permuted phenotypes, and compute *d*_*l*_ for each pathway. We then apply the following weight adjustment

$$\begin{array}{ll} w_{l}^{\left(\tau +1\right)}=w_{l}^{\left(\tau \right)}\left[1-\text{sign}\left(d_{l}\right)\left(\eta -1\right)L^{2}d_{l}^{2}\right] & 0<\eta <1\text{,}\;l=1\text{,…, }L \end{array}$$

where the parameter *η* controls the maximum amount by which each *w*_*l*_ can be reduced in a single iteration, in the case that pathway $G_{l}$ is selected with zero frequency. The square in the weight adjustment factor ensures that large values of |*d*_*l*_| result in relatively large adjustments to *w*_*l*_. Iterations continue until convergence, where $\sum_{l=1}^{L}|d_{l}|<\epsilon$.

Even when relatively few SNPs or genes are associated with the phenotype, we can expect multiple pathways to harbour genetic effects since many SNPs and genes overlap multiple pathways. Where more than one pathway is selected by the model, we therefore expect that pathway selection probabilities will not be uniform, since the presence of overlapping SNPs means that pathways are not independent. Instead, selection probabilities will reflect the pattern of overlaps corresponding to the distribution of causal SNPs (or spurious associations under the null). This non-uniform distribution of selection probabilities is to be expected and is in fact desirable, since a signal corresponding to causal SNPs or genes should be captured in each and every pathway that contains them. We have shown in extensive simulation studies, that where more than one pathway is selected, the weight tuning process described above leads to substantial gains in both sensitivity and specificity when identifying causal pathways (Silver and Montana, 2012).

Estimates for **b** and **a** respectively represent the first (rank 1) latent factors that are expected to capture the strongest signal of association between gene pathways and the phenotype. In principle, it is possible to capture further latent factors of diminishing importance, by iteratively repeating the procedure described above, after regressing out the effects of previous factors (Vounou et al., 2010). With PsRRR, the estimation of further ranks is complicated by the need to recalibrate the group weights at each step, and by the typically large number of SNPs in selected pathways. For this reason we consider only the first latent factor in this study.

### Pathway, gene and SNP ranking

#### Pathway ranking

With most variable selection methods, a choice for the regularisation parameter, λ, must be made, since this determines the number of variables selected by the model. Common strategies include the use of cross validation to choose a λ value that minimises the prediction error between training and test datasets (Hastie et al., 2008). One drawback of this approach is that it focuses on optimising the *size* of the set, $\hat{C}$, of selected pathways (more generally, selected variables) that minimises the cross validated prediction error. Since the variables in $\hat{C}$ will vary across each fold of the cross validation, this procedure is not in general a good means of establishing the importance of a unique set of variables (Vounou et al., 2011). Alternative approaches, based on data resampling or bootstrapping have been demonstrated to improve model consistency, in the sense that the ‘true’ variables are selected with a high probability (Bach, 2008; Meinshausen and Bühlmann, 2010). We adopt a resampling strategy, in which we calculate pathway selection frequencies by repeatedly fitting the model over *B* subsamples of the data, at a fixed value for λ. With this approach, which in some respects resembles the ‘pointwise stability selection’ strategy of Meinshausen and Bühlmann (2010), selection frequencies provide a direct measure of confidence in the selected pathways in a finite sample.

We denote the set of selected pathways at subsample *b* by

$${\hat{C}}^{\left(b\right)}=\left\{l:{\hat{\beta }}_{l}^{\left(b\right)}\neq 0\right\}\;b=1\text{,…, }B$$

where **b**_*l*_^(*b*)^ is the estimated SNP coefficient vector for pathway *l* at subsample *b*. The selection probability for pathway *l* measured across all *B* subsamples is then

$$\pi_{l}^{\mathrm{path}}=\frac{1}{B}\sum_{b=1}^{B}I_{l}^{\left(b\right)}\;l=1\text{,…, }L$$

where the indicator variable, *I*_*l*_^(*b*)^ = 1 if $l\in {\hat{C}}^{\left(b\right)}$, and 0 otherwise. Pathways are ranked in order of their selection probabilities, *π*_*l*_1__^*path*^≥,…, ≥ *π*_*l*_*L*__^*path*^.

#### SNP and gene ranking

The PsRRR model is designed to identify important pathways which may contain multiple genetic markers with varying effect sizes. However, it is still interesting to establish which SNPs and genes are most predictive of the response amongst those mapped to the set ${\hat{C}}^{\left(b\right)}$ of selected pathways at subsample *b*. Note that these are not necessarily the SNPs and genes that are driving the selection of any particular pathway in the PsRRR model.

To rank SNPs and genes, we perform a second level of variable selection using sRRR with a lasso penalty (Vounou et al., 2011). We first form the reduced (*N* × *Z*^(*b*)^) matrix $X_{{\hat{C}}^{\left(b\right)}}$, with columns $\left\{X_{j}:j\in \cup_{l\in {\hat{C}}^{\left(b\right)}}G_{l}\right\}$ corresponding to all SNPs in pathways selected at subsample *b*. Sparse estimates for the corresponding SNP coefficient vector, ***β***, and rank-1 phenotype vector ***α*** then satisfy the equivalent of Eq. (8) with a lasso penalty, namely

$$\hat{\beta },\hat{\alpha }=\underset{\beta ,\alpha }{\arg\min}\;\left\{\frac{1}{2}\left(-2\alpha Y^{\prime }X_{{\hat{C}}^{\left(b\right)}}\beta +\alpha \alpha^{\prime }\beta^{\prime }X_{{\hat{C}}^{\left(b\right)}}X_{{\hat{C}}^{\left(b\right)}}\beta \right)+\text{λ}{\left\|\beta \right\|}_{1}\right\}\text{.}$$

We denote the set of SNPs selected at sample *b* by ${\hat{S}}^{\left(b\right)}$, and further denote the set of selected genes to which the SNPs in ${\hat{S}}^{\left(b\right)}$ are mapped by *ϕ*^^^^(*b*)^ ⊂ Φ, where *Φ* = {1,…, *G*} is the set of gene indices corresponding to all *G* mapped genes. Using the same strategy as for pathway ranking, we obtain an expression for the selection probability of SNP *j* across *B* subsamples as

$$\pi_{j}^{\mathrm{SNP}}=\frac{1}{B}\sum_{b=1}^{B}I_{j}^{\left(b\right)}$$

where the indicator variable, *I*_*j*_^(*b*)^ = 1 if $j\in {\hat{S}}^{\left(b\right)}$, and 0 otherwise. A similar expression for the selection probability for gene *g* is

$$\pi_{g}^{\mathrm{gene}}=\frac{1}{B}\sum_{b=1}^{B}I_{g}^{\left(b\right)}$$

where the indicator variable, *I*_*g*_^(*b*)^ = 1 if $g\in {\hat{\phi }}^{\left(b\right)}$, and 0 otherwise. SNPs and genes are then ranked in order of their respective selection frequencies.

### Computational issues

All computer code is written in the open source Python programming language, using Numpy and SciPy modules which are optimised for efficient operation with large matrices. Execution of the PsRRR estimation algorithm nonetheless presents a considerable computational burden, both in terms of processor time and memory use. We therefore implement a number of strategies designed to increase computational efficiency (see Silver and Montana, 2012 for details). We use a Taylor approximation of the group penalty that avoids the need for computationally intensive numerical search methods (Breheny and Huang, 2009; Friedman et al., 2010). In addition, we use an ‘active set’ strategy (Roth and Fischer, 2008; Tibshirani et al., 2010), that identifies a subset of pathways that are more likely to be selected by the model at a given λ. Model estimation then proceeds with this reduced set, followed by a final check to ensure that no other pathways should have been included in the active set in the first place. Depending on the choice of λ, this can lead to substantial gains in computational efficiency and a large reduction in memory requirements, resulting from the very much reduced size of **X** in *Ω*(**a**, **Y**, **X**, λ).

The need to fit a large number of PsRRR models over multiple subsamples of the data for pathway ranking presents another major computational bottleneck. However, the fact that each subsample is generated entirely independently presents an opportunity for performing multiple model fits in parallel. We implement such a strategy using a computer cluster, in which a single client node distributes subsamples across 40 CPU cores. Parallel computations and client–server communication are implemented in Parallel Python (http://www.parallelpython.com/). The reduction in computation time due to parallelisation is considerable. For example, in the AD study described in this paper, total execution time (excluding weight tuning) with *B* = 1000 subsamples was 6 1/2 h, whereas total execution time if each job were run separately would be approximately 10 1/2 days.

## Results

### AD associated phenotypes

**Fig. 5:**
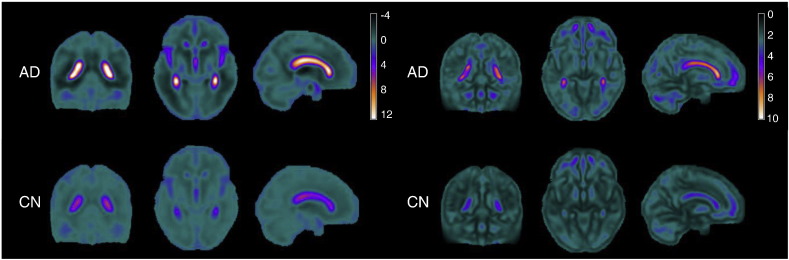
Sample mean *(left)* and standard deviation *(right)* of slope coefficients for the 2 subject groups. Slope coefficients represent a linear approximation of change in brain volume over time. Scales represent 10 × percentage change in voxel volume per year, so that for example a slope coefficient of 12 (white areas in left hand plot) is equivalent to an average yearly increase in voxel volume of 1.2 %.

An imaging signature characteristic of AD was created using the procedure described in the Phenotype extraction section. We begin by computing a linear least-squares fit of the longitudinal structural change across 3 time points at each voxel. An illustration of average slope coefficients, and their variation between subjects, is shown in Fig. 5. Increased expansion of ventricular volumes is clear in all subjects, but this increase is most marked in AD patients, where ventricular volumes expand by an average of 1.2% per year (white regions in left hand part of Fig. 5). AD patients also show the most variation in structural change over time.

**Fig. 6:**
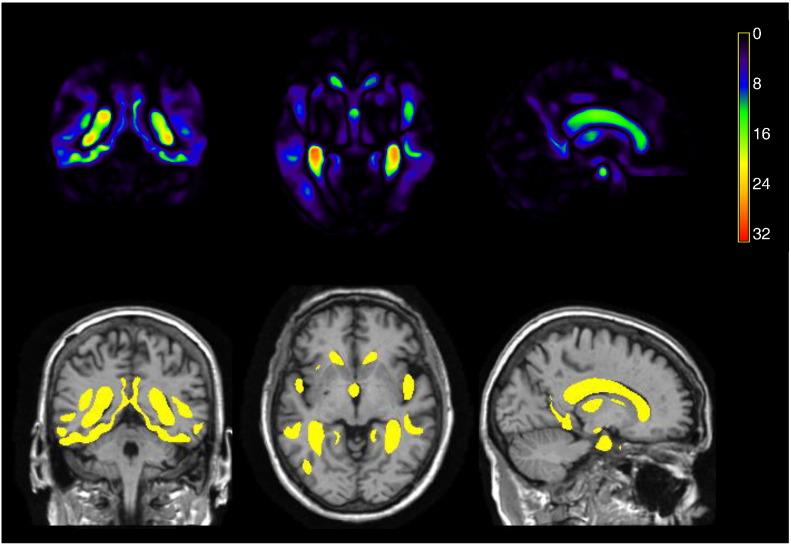
Imaging signature characteristic of AD. *Top*: Statistical image showing p-values (− log _10_ scale) obtained from an ANOVA on the linear structural change over 3 time points, corrected for age and sex, to discriminate between AD and CN subjects. *Bottom*: The final set of *Q* = 148, 023 selected voxels with p-values exceeding a Bonferroni-corrected threshold *α*_*B*_ = 0.05/2153231, (− log_10_ *α*_*B*_ = 7.6) are highlighted in yellow.

**Fig. 7:**
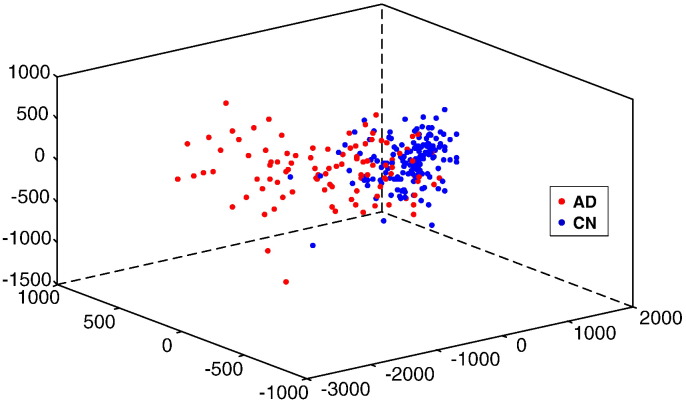
3D multi-dimensional scaling plot illustrating the spread of imaging signatures across ADs and CNs. Imaging signatures correspond to selected voxels only.

A statistical image showing the corresponding ANOVA p-values, a measure of the extent to which each voxel is able to discriminate between ADs and CNs, is shown in the top row of Fig. 6. From the *Q*^∗^ = 2, 153, 231 voxels in this image, we extract a final set of *Q* = 148, 023 voxels whose p-values exceed a Bonferroni-corrected threshold of 0.05/*Q*^∗^. This final set of voxels that is most discriminative between ADs and CNs is highlighted in yellow in the bottom row of Fig. 6. These *Q* voxels constitute the phenotype for each subject used in the study. We provide a further indication of the discriminatory power of the selected voxels by visualising the Euclidean distances between subjects using the selected voxels in a 3D multi-dimensional scaling plot in Fig. 7. The relatively small overlap between CD and AD subjects indicates that our chosen disease signature is indeed discriminative between the two groups. As expected we also see evidence of greater variability in the AD group, compared with CN.

### Pathway, SNP and gene rankings

We use the PsRRR algorithm described in the Pathways sparse reduced-rank regression section to identify KEGG pathways associated with the AD-discriminative longitudinal phenotypes described in the preceding section. Pathways are ranked in order of importance using the resampling strategy described in the Pathway, gene and SNP ranking section, with *B* = 1000 subsamples. We use λ = 0.8 λ_*max*_, which results in the selection of an average of 7 pathways at each subsample (min 1, max 15, SD = 2.3). Pathway ranking results are presented in Table 3.

SNPs and genes are ranked using sRRR with a lasso penalty on the SNP coefficient vector, as described in the Pathway, gene and SNP ranking section. Lasso selection is performed on pathways selected at each subsample in the pathway analysis described above, so that once again *B* = 1000. The number of SNPs, *Z*^(*b*)^, included in the lasso model at subsample *b* varies according to the number and size (in terms of the number of mapped SNPs) of selected pathways. *Z*^(*b*)^ ranges from a minimum of 227, to a maximum of 19,642 (mean = 8400; SD = 3000). As with pathway ranking, we use λ = 0.8 λ_*max*_, which results in the selection of an average of 11.5 SNPs at each subsample (min 1, max 56, SD = 11.7). SNP and gene ranking results are presented in Table 4.

We first consider the pathway ranking results in Table 3. Under the null, where there is no association between phenotypes and genotypes, and with a single pathway selected by the model at each subsample, the expected pathway selection frequency distribution is uniform, with, *π*_*l*_^*path*^ = 1/185 ≈ 0.005. With an average of 7 pathways selected at each subsample, as is the case here, and assuming pathways are independent, the corresponding pathway selection frequency distribution under the null is also uniform, with, *π*_*l*_^*path*^ = 7/185 ≈ 0.038. However, as explained in the Pathways sparse reduced-rank regression section, the presence of SNPs (and genes) overlapping multiple pathways means that where more than one pathway is selected at each subsample, the selection frequency distribution will depend on the distribution of causal SNPs and genes, and will not be uniform. For this reason the figure of 0.038 should be seen only as a guide threshold to signify pathway importance, and while we report pathway selection frequencies, *π*_*l*_^*path*^, our main focus is on pathway rankings. To aid interpretation of pathway rankings, for each pathway, we list those genes in the pathway that are ranked in the top 30 genes, selected by lasso selection (in Table 4).

**Fig. 8:**
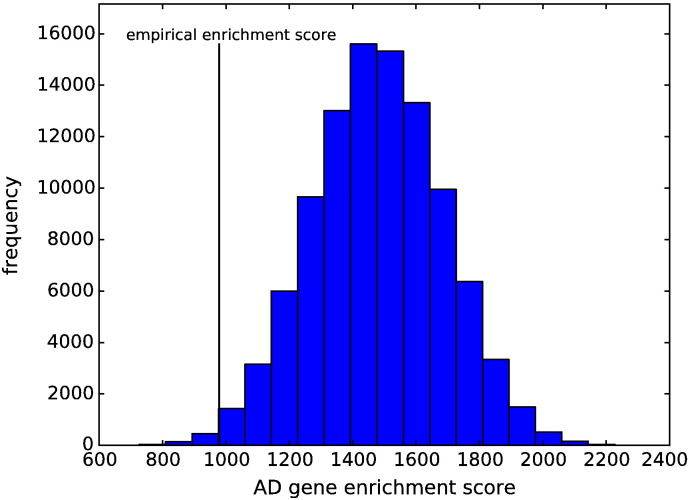
Measure of extent to which genes previously linked to AD are enriched in highly-ranked pathways. The histogram shows the distribution of AD gene enrichment scores obtained when permuting pathway rankings 100,000 times. The vertical black line indicates the observed AD gene enrichment score using the true pathway rankings obtained in the study. From this we derive a p-value indicating the probability that the empirical AD gene enrichment score could arise by chance as p = 0.0051. AD-linked genes are those identified in Braskie et al. (2011).

In the final column of Table 3 we list genes in the top ranked pathways that have previously been linked to AD in the review by Braskie et al. (2011). Both the number of such genes affecting phenotypes in this study, and the extent to which these genes may drive pathway selection are unknown. It is nevertheless interesting to consider whether these genes are significantly enriched amongst high-ranking pathways. To do this we calculate an average ranking for each ‘AD gene’ by taking the average rank achieved by all pathways containing the gene in question. We then derive an AD gene enrichment score by summing average AD gene ranks across all AD genes. A lower score thus indicates that pathways containing AD genes tend to be ranked high. We compare this empirically derived score with the distribution of scores obtained by permuting pathway rankings 100,000 times. The null distribution of this enrichment score (obtained by permutation), and the empirically observed value are compared in Fig. 8. Finally, we compute a p-value for the null hypothesis that the empirically observed enrichment score has arisen by chance, as the proportion of enrichment scores obtained through permutation that are lower than the observed value. This gives a value p = 0.0051, indicating that AD genes are highly over-represented amongst top ranking pathways, compared to what would be expected by chance.

## Discussion

We describe a method for the identification of gene pathways associated with a multivariate quantitative trait (MQT). Here, we extend previous work modelling a univariate response, where we showed that a multilocus, group-sparse modelling approach can demonstrate increased power to detect causal pathways, when compared to conventional approaches that begin by modelling individual SNP-phenotype associations (Silver and Montana, 2012). We apply our method in an AD gene pathway study using imaging endophenotypes, but our method is not restricted to the case of biological pathways or imaging phenotypes, and can be applied to any data in which we seek to identify sparse groups of predictors affecting a multivariate response.

In any method modelling effects on an MQT, the use of a multivariate disease signature that is characteristic of the disease under investigation is important. This is especially so in the case of high-dimensional imaging phenotypes, where a poorly characterised imaging signature with low signal to noise ratio may show no advantage over a simple ROI average-based approach (Vounou et al., 2011). In this study we extract an AD imaging phenotype that is highly discriminative of subjects with the disease, compared to controls, by excluding voxels at which the fitted slopes, measuring structural change over 3 time points, are not significantly different between the two groups. The subsequent pathway and gene mapping stages will clearly depend on the particular choice of phenotype, so that a different choice of phenotype may well highlight different genetic effects. An analysis of the sensitivity of our gene mapping procedure to the choice of phenotype is however beyond the scope of the present study. We note that implicit in our overall strategy is the assumption that our imaging phenotype is indeed characteristic of AD-related structural change in the general population. Ideally we would therefore like to validate these results using an independent dataset. However, at the time of writing no other datasets with similar imaging endophenotypes were available.

We use a resampling strategy to rank pathways by selection frequency across multiple *N*/2 subsamples of the data. This strategy is designed to provide a robust measure of the relative importance of individual pathways in a finite sample (Silver and Montana, 2012). In some respects our approach resembles the ‘pointwise stability selection’ strategy proposed by Meinshausen and Bühlmann (2010). For the latter, a theoretical bound for determining a selection frequency threshold that controls the expected number of false positives has been derived. However, this rests on the assumption that selected variables are independent, which is not the case here, since the variables under selection are groups of variables (pathways) that are functionally related, and overlap in terms of the genes that they contain. Indeed a feature of our method is that we expect to identify multiple, possibly interacting pathways where the signal is strong.

Of the top-ranking pathways identified in our study (see Table 3), functions associated with many of the top 10 ranked pathways have been linked to aspects of AD biology described in the literature. Beginning with the top 2 ranked pathways, numerous studies suggest links between disruption to the insulin signalling pathway and AD (Biessels and Kappelle, 2005; de la de la Monte and Wands, 2005; Liao and Xu, 2009; Liu et al., 2011; Steen et al., 2005), and to the role of vascular smooth muscle dysfunction in AD-associated neurodegeneration (Zlokovic, 2011). Other functions previously associated with AD biology among high-ranking pathways include those related to focal adhesion, gap junctions, chemokine signalling and phosphatidylinositol signalling (Caltagarone et al., 2007; Huber et al., 2001; Kim et al., 2003; Nakase and Naus, 2004; Ravetti et al., 2010; Xia and Hyman, 1999).

In order to better elucidate which genes may be driving pathway selection, we performed a follow up analysis designed to identify SNPs and genes in selected pathways that are separately associated with the phenotype (see Table 4). Since these gene (and associated SNP) rankings are derived from lasso selection of all SNPs within selected pathways, irrespective of their ‘group’ structure within pathways, they are expected to capture larger, independent signals of association, and not necessarily all the salient signals within a particular pathway that may be driving pathway selection. In particular, the group lasso is designed to detect distributed signals that may not be highlighted using lasso selection. From this analysis, it is clear that the lipid kinase genes *PIK3R3/PIK3CG*, and the calcium-activated, phospholipid-dependent genes *PRKCA/PRKCB* are important in driving selection of many pathways in the top 30 ranks. All these genes have previously been linked in gene expression studies with *β*-amyloid plaque formation in the AD brain (Liang et al., 2008). Aside from the previously validated AD endophenotype-related genes *TOMM40*, *CR1* and *APOE* (Biffi et al., 2010; Lambert et al., 2009; Shen et al., 2010), other genes occurring in the top 10 ranking pathways, include *ADCY2*, *ACTN1*, *ACACA* and *GNAI1*, all of which have been associated with AD related changes in hippocampal gene expression (Ravetti et al., 2010; Taguchi et al., 2005, supporting information). Along with *APOE* and *TOMM40*, *ADCY2* was also highlighted in a previous study searching for SNPs associated with AD-associated structural change (Vounou et al., 2011). This latter study was on the same ADNI cohort, but unlike the current study it was not pathway-driven, and used phenotypes describing structural change measured at a single time point (relative to baseline) only.

The major AD risk and phenotype-related gene *APOE*, and risk allele *APOEϵ*4 are respectively ranked 19 and 9. In our study the *APOE* gene maps to a single pathway, the KEGG Alzheimer's disease pathway, and this pathway is selected in ≈ 13 % of subsamples. Notably, in all subsamples in which the KEGG Alzheimer's disease pathway is selected, the *APOEϵ*4 allele is the sole selected SNP, confirming the known large marginal effect of this allele on AD phenotypes. The higher ranking of the *APOEϵ*4 SNP, relative to the *APOE* gene, reflects the fact that this SNP also maps to the *TOMM40* gene, which occurs in a number of other pathways selected by the model. This may affect the Alzheimer pathway's ranking, as may the fact that selection of this pathway is driven by the presence of this single, strong *APOEϵ*4 signal, and as explained above, the model is designed to identify distributed signals across a pathway.

In principle our method enables the voxel-wise mapping of pathway effects across the brain, through the analysis of the phenotype coefficient vector **a**, although we do not report this here. We note that the use of an additional regularisation penalty on **a** to enforce the sparse selection of important voxels, would make an interesting extension to our method, by highlighting specific voxels or regions with a putative association with high ranking causal pathways. Suitable sparse regression models include the lasso and the elastic net (Carroll et al., 2009), although both would require the tuning of addition regularisation parameters.

Our model rests on a number of assumptions, and as a consequence will fail to detect a number of different association signals. For example, while our model implicitly accommodates the fact that SNPs and genes interact within functional pathways, we do not explicitly model interaction effects. Also, we make the simplifying assumption that voxel-wise measures of atrophy are uncorrelated. In reality, the phenotype will exhibit a complex correlation structure which will affect the association signal. Vounou et al. (2010) have demonstrated that even under this simplifying assumption, significant gains in power can be achieved by modelling a multivariate phenotype, compared to a mass univariate modelling approach. Finally, our model is founded on the assumption that causal SNPs tend to accumulate within functional pathways, and as such is not designed to identify significant marginal effects, as evidenced by its failure to rank the high-risk *APOE* gene highly. For this last reason, any pathway analysis should be seen as being complementary to conventional GWAS approaches.

To the best of our knowledge, there are few other multilocus pathway methods, and none are able to accommodate a multivariate, quantitative phenotype. While a methodological study comparing the various approaches would be interesting, as has been noted by others, a lack of benchmark datasets with validated pathways makes comparison between methods difficult (Chen et al., 2010; Khatri et al., 2012).

The present study demonstrates some of the limitations of pathway studies in general. Many genes previously implicated in AD do not map to known pathways in our study, so that these genes and their associated SNPs, many of which are well validated, are excluded. This further reinforces the point that pathway studies should be seen as complementary to studies searching for single markers, since a significant part of the known AD-associated genetic signal is missing. The relative sparsity of gene-pathway annotations reflects the fact that our understanding of how the majority of genes functionally interact is at an early stage. As a consequence, annotations from different pathway databases often vary (Soh et al., 2010), and in any case are undergoing rapid change.

## Figures and Tables

**Table 1:** Available scans for the ADNI-1 dataset (downloaded on February 28, 2011).

	Screening	6 mo	12 mo	24 mo
AD	200	165	144	111
CN	232	214	202	178
Total	432	379	346	289


At screening:			
Group	Age (years)	N male	N female
AD	75.7 ± 7.7	103	97
CN	76.0 ± 5.0	120	112

**Table 2:** AD genes included in this study. 12 out of 30 genes previously implicated with AD (Braskie et al., 2011) that are included in this study are listed in the left hand column. These are genes that (a) map to a KEGG pathway and (b) have a genotyped SNP within 10 kbp. The right hand column shows neighbouring genes that map to one or more SNPs mapping to the respective AD implicated gene.

Implicated gene	Mapped genes in study
*TOMM40*	*TOMM40 APOE PVRL2*
*ACE*	*ACE*
*EPHA4*	*EPHA4*
*CCR2*	*CCR2 CCR5*
*APOE*	*TOMM40 APOE PVRL2*
*FAS*	*FAS*
*CHRNB2*	*ADAR CHRNB2*
*EFNA5*	*EFNA5*
*LDLR*	*LDLR*
*CR1*	*CR1 CR2*
*GRIN2B*	*GRIN2B*
*IL8*	*IL8*

**Table 3:** Top 30 pathways, ranked by pathway selection frequency.

Rank	KEGG pathway name	*π*^*path*^	Size (# SNPs)	Lasso selected genes in pathway^1^	Known AD genes^2^ in pathway
1.	Insulin signalling pathway	0.524	1517	*HK2 PIK3R3 PIK3CG ACACA G6PC*	
2.	Vascular smooth muscle contraction	0.456	3236	*PRKCB ADCY8 ADCY2 PRKCA MYLK PLCB1*	
3.	Melanogenesis	0.331	1638	*PRKCB ADCY8 ADCY2 PRKCA GNAI1 WNT2 PLCB1*	
4.	Focal adhesion	0.232	4009	*PRKCB PRKCA PIK3R3 MYLK PIK3CG COL5A3 RELN ACTN1*	
5.	Gap junction	0.180	2350	*PRKCB ADCY8 ADCY2 PRKCA GNAI1 PLCB1*	
6.	Huntington's disease	0.155	1980	*PLCB1 DNAI2 UQCRH*	*GRIN2B*
7.	Purine metabolism	0.154	2896	*ADCY8 ADCY2 ALLC*	
8.	Pyruvate metabolism	0.153	456	*ACACA*	
9.	Propanoate metabolism	0.152	471	*ACACA*	
10.	Amyotrophic lateral sclerosis ALS	0.151	865	*TOMM40*	*TOMM40 GRIN2B*
11.	Chemokine signalling pathway	0.145	2769	*PRKCB ADCY8 ADCY2 PIK3R3 PIK3CG GNAI1 PLCB1 XCL1 ITK GNG2 GRK5*	*CCR2 IL8*
12.	Phosphatidylinositol signalling system	0.138	2067	*PRKCB PRKCA PIK3R3 PIK3CG DGKA DGKB PLCB1 DGKI*	
13.	Citrate cycle TCA cycle	0.137	210		
14.	Glycosphingolipid biosynthesis globo series	0.135	227		
15.	Alzheimer's disease	0.127	2500	*PLCB1 APOE UQCRH*	*APOE FAS GRIN2B*
16.	Complement and coagulation cascades	0.119	783	*CR1*	*CR1*
17.	Steroid biosynthesis	0.113	153		
18.	Jak stat signalling pathway	0.106	1311	*PIK3R3 PIK3CG*	
19.	ECM receptor interaction	0.104	1969	*COL5A3 RELN*	
20.	Tight junction	0.103	3332	*PRKCB PRKCA GNAI1 ACTN1 YES1*	
21.	Glycerolipid metabolism	0.102	877	*DGKA DGKB DGKI*	
22.	Calcium signalling pathway	0.096	5111	*PRKCB ADCY8 ADCY2 PRKCA MYLK PLCB1*	
23.	Toll like receptor signalling pathway	0.096	712	*PIK3R3 PIK3CG*	*IL8*
24.	Leishmania infection	0.090	620	*PRKCB CR1*	*CR1*
25.	Lysosome	0.089	1111		
26.	Fc gamma R mediated phagocytosis	0.080	1976	*PRKCB PRKCA PIK3R3 PIK3CG*	
27.	Neurotrophin signalling pathway	0.075	1689	*PIK3R3 PIK3CG*	
28.	Glycerophospholipid metabolism	0.071	1047	*DGKA DGKB DGKI*	
29.	Renal cell carcinoma	0.071	840	*PIK3R3 PIK3CG*	
30.	Wnt signalling pathway	0.070	2023	*PRKCB PRKCA WNT2 PLCB1*	

1 Top 30 ranked genes in this pathway, using lasso selection (see Table 4).

2 Previously identified AD genes in the pathway (see Table 2).

**Table 4:** Top 30 SNPs and genes, respectively ranked by SNP and gene selection frequency, using lasso sRRR. Note the APOE gene is selected at a lower frequency than the *APOE*ϵ4 since the allele is often selected in a pathway where it is mapped to the TOMM40 gene only.

Rank	SNP RANKING			GENE RANKING		
SNP	*π*^*SNP*^	Mapped gene(s)	Gene	*π*^*gene*^	# mapped SNPs
1	rs4788426	0.451	*PRKCB*	PRKCB	0.451	73
2	rs11074601	0.429	*PRKCB*	ADCY8	0.411	69
3	rs263264	0.411	*ADCY8*	ADCY2	0.392	106
4	rs13189711	0.392	*ADCY2*	HK2	0.302	28
5	rs680545	0.302	*HK2*	PRKCA	0.290	99
6	rs4622543	0.290	*PRKCA*	PIK3R3	0.267	9
7	rs9896483	0.274	*PRKCA*	MYLK	0.234	24
8	rs1052610	0.267	*PIK3R3*	PIK3CG	0.207	9
9	*APOE*ϵ4	0.251	*TOMM40 APOE*	COL5A3	0.174	14
10	rs1254403	0.234	*MYLK*	GNAI1	0.167	22
11	rs4730205	0.207	*PIK3CG*	ACACA	0.164	23
12	rs889130	0.174	*COL5A3*	G6PC	0.163	6
13	rs6973616	0.167	*GNAI1*	DGKA	0.160	3
14	rs9906543	0.164	*ACACA*	CR1	0.154	21
15	rs2229611	0.163	*G6PC*	TOMM40	0.152	6
16	rs10876862	0.160	*DGKA*	WNT2	0.137	12
17	rs772700	0.160	*DGKA*	DGKB	0.131	200
18	rs12734030	0.154	*CR1*	PLCB1	0.128	218
19	rs11117959	0.154	*CR1*	APOE	0.127	4
20	rs650877	0.154	*CR1*	RELN	0.117	160
21	rs11118131	0.154	*CR1*	DGKI	0.112	49
22	rs6691117	0.142	*CR1*	ACTN1	0.110	41
23	rs677066	0.142	*CR1*	ALLC	0.108	18
24	rs2239956	0.137	*WNT2*	XCL1	0.086	7
25	rs4719392	0.131	*DGKB*	ITK	0.084	27
26	rs6077420	0.128	*PLCB1*	DNAI2	0.077	16
27	rs7777178	0.126	*DGKB*	GNG2	0.076	31
28	rs12699607	0.122	*DGKB*	GRK5	0.074	56
29	rs7796440	0.122	*DGKB*	UQCRH	0.071	2
30	rs1872837	0.120	*HK2*	YES1	0.068	11

## Box 1

Ω(**a**, **Y**, **X**, λ): GL estimation algorithm using BCD.

**Table:**

1.	**b** ← 0
	block coordinate descent
2.	**repeat**
3.	**for***l* = 1, 2,…, *L*
4.	$r_{l}\leftarrow Ya^{\prime }-\sum_{k\neq l}X_{k}b_{k}$
5.	**if**${\left\|{X^{\prime }}_{l}r_{l}\right\|}_{2}\leq \text{λ}w_{l}$
6.	*b*_*l*_ ← 0
7.	**else**
	coordinate descent within block
8.	**repeat**
9.	**for***j* = *l*_1_,…, *l*_*S*_*l*__
10.	$r\leftarrow Ya^{\prime }-Xb$
11.	$b_{j}\leftarrow \frac{{X^{\prime }}_{j}r+b_{j}}{1+\frac{\lambda w_{l}}{{\left\|b_{l}\right\|}_{2}}}$
12.	**until b**_*l*_ converges
13.	**until b** converges

## Box 2

Rank-1 PsRRR estimation algorithm using coordinate descent.

**Table:**

1.	**a** ← 1/‖1‖_2_
2.	**repeat:**
3.	λ ← *γ*λ_*max*_, where ${\text{λ}}_{\max}=\min_{\text{λ}}\left\{\text{λ}:{\left\|X_{l}^{T}Ya^{\prime }\right\|}_{2}=\text{λ}w_{l}\text{,}\;l=1\text{,…, }L\right\}$
4.	**b** ← *Ω*(**a**, **Y**, **X**, λ) (from Box 1)
5.	**b** ← **b**/‖**b**‖_2_ (normalise)
6.	$a\leftarrow \frac{b^{\prime }X^{\prime }Y}{b^{\prime }X^{\prime }Xb}$
7.	**a** ← **a**/‖**a**‖_2_ (normalise)
8.	**until b** and **a** converge
